# Application of variance components to the identification of determinants of modern contraceptive use in the Tanzania demographic and health survey data

**DOI:** 10.1186/s12889-022-13636-5

**Published:** 2022-07-05

**Authors:** Oliva Safari Donni, Dunstan Raphael Bishanga, Isambi Sailon Mbalawata

**Affiliations:** 1 Medical Teams International(MTI) Tanzania, Department of Monitoring, Learning and Evaluation, P O Box 1, Kibondo, Tanzania; 2 School of Public Health and Social Sciences, Muhimbili University of Health and Allied Sciences, P O Box 65001, Dar es Salaam, Tanzania; 3 AIMS-NEI Global Secretariat, Gasabo Kacyiru - Kamatamu, KG590 ST, Kigali, Rwanda

**Keywords:** MCU, TDHS, Variance component, Random coefficient, Nested structure

## Abstract

**Background:**

Over time, demographic and health survey (DHS) data remain valuable to examine variables relating to nationally representative population outcomes for low- and middle-income countries. In Tanzania, there are very limited DHS-based studies on the uptake of Modern Contraceptive Use (MCU). Present studies have focused on measurements at the level of individuals, yet research has shown that MCU variations exists at other levels within populations. In this study, we use a variance component modelling approach to explore variation in MCU at primary sampling unit (PSU) and regional levels while considering survey sample weights.

**Methods:**

Using DHS data from 2016–2017 in Tanzania, we study different variance structures and the respective variation on MCU in a sample of 5263 Women of Reproductive Age (WRA) defined as between the ages of 15–49 years. First, a single variance component was used, followed by its extension to a random coefficient model and we tracked changes in the models.

**Results:**

There was an influence of random variations on MCU on the levels of populations much explained by PSU-level clustering than region. On the fixed part, age of a woman, husband education level, desire to have children, and exposure to media and wealth tertiles were important determinants for MCU. Compared to WRA in 15–19 years, the odds of MCU among middle aged women (20–29 and 30–39 years) were 1.94 (95%CI:1.244–3.024) and 2.28 (95%CI:1.372–3.803). Also, increases in media exposure and middle and rich wealth tertiles women led to higher odds for MCU. We also found the presence of random effects influence of wealth tertiles levels on MCU.

**Conclusion:**

This study highlighted the utility of accounting for variance structures in addressing determinants of MCU while using DHS national level data. Apart from MCU, the DHS data have been widely applied to examine other variables pertaining to public health issues. This approach could be considered a better modelling technique for the DHS studies compared to traditional survey approaches, and to guide hierarchical population-based interventions to increase MCU.

## Background

There is considerable literature on MCU in low-and middle-income countries (LMIC). Increasing the uptake of MCU remains crucial in addressing maternal and child health services despite switching from Millennium to Sustainable Developments Goals [1–3]. The importance of MCU over natural methods is primarily seen in preventing unplanned pregnancies, with global reductions from 43% in 1995 to 40% in 2012. This was influenced by increased demand for family planning, as demonstrated by the increase in demand for MCU from 68 to 78%. MCU also helps in control of fertility rates through the practice of child spacing.

A recent global report shows that MCU has increased from 54% in 1990 to 57% in 2015 [2]. In Sub-Saharan African (SSA) usage remained persistently low; around 23% and 24% respectively. The same report showed a higher usage rate in high income countries (HIC), with 80% and above in the UK and China, and 75% in North America, and how in many developed countries maternal and child health status have improved [4, 5]. However, in Tanzania, 24% of women of reproductive age are facing a crucial unmet need for family planning. This is twice the world average (12%) and stems from a low (32%) use of contraceptives, particularly modern contraceptives [6]. Also 50% of maternal mortalities within the country are attributed to low and ineffective uptake of MCU [6]. In addition, Tanzania is among the leading African countries in terms of fertility rate (4 children per woman). Both maternal mortality and fertility rates could be controlled by effective MCU [7, 8]. In recognizing the need for MCU, the Government of Tanzania imposed several strategies to double the number of contraceptive users by the year 2015. This included providing high-quality, accessible, acceptable, and affordable Family Planning (FP) services for young people, strengthening the supply chain for FP commodities, increasing male involvement in FP issues and a mass media campaign [6]. The uptake of MCU remains extremely low in Tanzania and there is a marked regional variation from 6.8% in Southern Zanzibar to 50.8% in Ruvuma [6, 9].

Using DHS data, studies have reported on various covariates associated with MCU, including education levels among women of reproductive health, age levels, exposure to media and wealth tertiles levels [10, 11]. A Tanzania demographic and health survey(TDHS)-based study identified factors associated with MCU including difference in age levels among partners, partners levels of education and women empowerment [12]. However, other studies among different populations in Tanzania reported similar characteristics associated with MCU [13–16]. Most of these studies focus on small-scale samples within limited target populations (secondary school students, university populations, and HIV groups), that are less representative of overall WRA within the country but only to the specific group on which the study was conducted. Furthermore, when the TDHS data are used to study WRA then modelling approaches often deployed unweighted sampling modelling methods. Despite a study reporting that survey data weighting has slight inferential difference with unweighted data [17], it is still important that the modelling of DHS should consider not only sample weighting, but also account for the hierarchical nature of the data resulting from multistage sampling. Also, MCU uptake may vary from point to point with respect to individuals’ characteristics, thus it is important for public health research to consider statistical approaches indicating evidence-based variations. Therefore, this analysis aimed to determine the proportion of variability in MCU encountered at different hierarchical levels and how these affected estimates of MCU. Specifically, we aimed to

I. estimate the magnitude of heterogeneity introduced by PSUs and region in MCU.
II. identify determinants of MCU while accounting for variance structures.
III. determine the effect of additional random covariate on MCU.
IV. draw lessons for public health policy, applications, and practices.

## Methods

### Study settings

This study used data from the 2015/2016 TDHS available from www.dhsprogram.com, which are normally conducted every 5 years and include health related variables of the populations under study. The sample selections were based on two stage sampling with enumeration areas (EAs) which were streets in urban centers or villages in rural areas as the PSUs, followed by selection of households which contains WRA. More details on DHS-variables can be obtained from the DHS manual [18].

A total of 13,266 WRA were sampled, 5077 were dropped as they were not married, 1862 women who desired children in next 2 years were omitted, 188 infecund women were dropped, and 965 women who were pregnant were also removed from study as they were not recommended for MCU. This left 5263 (weighted sample) WRA eligible for the MCU study who were nested within 568-PSUs nested within 55-regions.

### Variables

The TDHS program usually collect a lot of information including the status of MCU by any modern method. The dependent variable was obtained by a dichotomization process with 1 for women who used one of the following; injectable, pills, sterilizations, Inter-Uterine Device (UID), condoms and lactation amenorrhea and 0 otherwise [6]. Covariates used include woman’s age group in years (15–19, 20–29, 30–39, 40–49), woman’s educational level (never to schools, primary, secondary +), exposure to media: radio, television, newspaper (not exposed at all, exposed to at least one, exposed to at least two, exposed to all), wealth tertiles (poor, middle, rich), urban–rural place of residence, and parity (no child, 1 child, 2 children, 3 + children) [6, 10, 11, 19, 20].

### Statistical considerations

#### The variance component modelling approach to MCU

Variance components were used in 1918 by Fisher in genetics studies and later in 1931 by Tippest in sampling. The approach has increased in popularity in a variety of fields such as agriculture; biology for laboratory trials and medicine; education, engineering, and experimental and survey or panel data to address many sources contributing to variations of a characteristic or a variables or process [21, 22]. However, the approach is commonly used in connection with clustering, nested models, mixed effects models, multilevel models, and hierarchical or random effect models [23–25].

Following Leyland and Goldstein [22], consider a grouped or hierarchical data structure from TDHS in which individuals are sampled from 608 PSUs across the whole country, say 608 PSUs representing a whole population of WRA in the country. That is, individual $\mathrm{i}$ (i = 1,2,3……, ${\mathrm{i}}_{\mathrm{k}}$), ${\mathrm{i}}_{\mathrm{k}}$= 5263 (the total sample size) sampled from PSU (villages and streets) $\mathrm{j}$ (j = 1,2, 3……, ${\mathrm{j}}_{\mathrm{q}}$), ${\mathrm{j}}_{\mathrm{q}}$= 608. Our interest might be on the population forming a sample of PSUs than PSUs themselves, thus PSUs are considered as a random sample from an infinite hypothetical population of PSUs. Infinite hypothetical in a sense that the PSUs’ population is unknown unlike the population defining individuals. To model such data, we need to consider two kinds of variation: that between individuals in the same PSU and between PSUs themselves, giving the following model expression:

1 $${\mathrm{Y}}_{\mathrm{ij}}={\upbeta }_{0\mathrm{j}}+ {\mathrm{u}}_{0\mathrm{j}}+{\mathrm{e}}_{\mathrm{ij}}$$

where, ${\mathrm{Y}}_{\mathrm{ij}}$ is the ${\mathrm{i}}^{\mathrm{th}}$ individual observation regarding MCU in the ${\mathrm{j}}^{\mathrm{th}}$ PSU, ${\upbeta }_{0\mathrm{j}}$ is given by ${\upbeta }_{0}+{\mathrm{u}}_{0\mathrm{j}}$ and defined as a random parameter of a population of quantities with mean value ${\upbeta }_{0}$. The ${\upbeta }_{0}$ describes the fixed part of the model. It is also called a random intercept which is the average value of ${\mathrm{Y}}_{\mathrm{ij}}$ when there are no covariates in a model or the mean value of MCU across PSUs with zero influence of predictors. ${\mathrm{u}}_{0\mathrm{j}}$ is the random component applying to all individuals in the ${\mathrm{j}}^{\mathrm{th}}$ PSU with mean value of 0 and variance ${\updelta }_{\mathrm{u}0}^{2}$. ${\mathrm{e}}_{\mathrm{ij}}$ is another random component for the ${\mathrm{i}}^{\mathrm{th}}$ woman in the ${\mathrm{j}}^{\mathrm{th}}$ PSU; its mean is 0 and variance ${\updelta }_{\mathrm{e}}^{2}$. ${\mathrm{u}}_{0\mathrm{j}}$ and ${\mathrm{e}}_{\mathrm{ij}}$ are random parameters with zero correlation between themselves, forming a random part of the model and partitioning the variance into two parts ${\mathrm{e}}_{\mathrm{ij}}$ and ${\mathrm{u}}_{0\mathrm{j}}$. The fact that the total variance is divided into multiple levels of ${\updelta }^{2}\mathrm{s}$ leads to the term *variance components* (two level variances). The two levels variances come from variances associated with the individuals and the PSUs respectively, allowing for variation in MCU across two levels. The two terms (${\mathrm{u}}_{0\mathrm{j}}$ and ${\mathrm{e}}_{\mathrm{ij}}$) both have expectations of zero;

2 $$\mathrm{E }({\mathrm{e}}_{\mathrm{ij}}) =\mathrm{ E }({\mathrm{u}}_{0\mathrm{j}})= 0$$

In this study, we model MCU as a binary variable coded as 1 for a user, and 0 for a non-user.

3 $${\mathrm Y}_{\mathrm{ij}}=\left\{\begin{array}{cc}&1\;\mathrm{if}\;\mathrm{an}\;\mathrm{individual}\;\mathrm{is}\;\mathrm a\;\mathrm{modern}\;\mathrm{contraceptive}\;\mathrm{user}\\0&\mathrm{otherwise}\end{array}\right.$$

Thus, expectation or conditional probability that there is MCU given a single predictor variable ${\mathrm{X}}_{1\mathrm{ij}}$, is defined as:

4 $$\mathrm{E }({\mathrm{Y}}_{\mathrm{ij}},{\mathrm{X}}_{1\mathrm{ij}}) ={\upbeta }_{0}+{\upbeta }_{1}{\mathrm{X}}_{1\mathrm{ij}}$$

Since (2) will be zero. This can be regarded as probability of success when ${\mathrm{Y}}_{\mathrm{ij}}$=1 given covariate ${\mathrm{X}}_{1\mathrm{ij}}$;

5 $$\mathrm{P }({\mathrm{Y}}_{\mathrm{ij}}=1/{\mathrm{X}}_{1\mathrm{ij}})=\mathrm{P }({\upbeta }_{0}+{\upbeta }_{1}{\mathrm{X}}_{1\mathrm{ij}}+{\mathrm{u}}_{0\mathrm{j}}+{\mathrm{e}}_{\mathrm{ij}})=1,{\mathrm{X}}_{1\mathrm{ij}}=\mathrm{P }({\mathrm{e}}_{\mathrm{ij}}-{\upbeta }_{0}-{\upbeta }_{1}{\mathrm{X}}_{1\mathrm{ij}}- {\mathrm{u}}_{0\mathrm{j}})$$

and equals to;

6 $$\mathrm{F }({\upbeta }_{0}+{\upbeta }_{1}{\mathrm{X}}_{1\mathrm{ij}}+{\mathrm{u}}_{0\mathrm{j}})={\uppi }_{\mathrm{ij }}(\;\mathrm{the}\;\mathrm{success}\;\mathrm{probability}-\;\mathrm{when}\;\mathrm{MCU}=1)$$

Equation (6) is defined as ${\uppi }_{\mathrm{ij}}$ which indicates the success probability function i.e. when there is MCU given exposure variable*.*${\pi }_{ij}$ and $1-{\uppi }_{\mathrm{ij}}$ define success and failure values, respectively for a binomial probability since there are two possibilities for MCU uptake; use (when there is MCU uptake) and non-use (when there is no uptake) respectively. From (4), we have a binary response variable which after adoption of the logistic link function providing the following models expression;

7 $$\mathrm{log }\left(\frac{{\uppi }_{\mathrm{ij}}}{{1-\uppi }_{\mathrm{ij}}}\right)={\upbeta }_{0}+{\upbeta }_{1}{\mathrm{X}}_{1\mathrm{ij}}+{\mathrm{u}}_{0\mathrm{j}}$$

8 $$\;\mathrm{or}\;\mathrm{else},\;{\uppi }_{\mathrm{ij}}=\frac{\mathrm{exp}\left({\upbeta }_{0}+{\upbeta }_{1}{\mathrm{X}}_{1\mathrm{ij}}+{\mathrm{u}}_{0\mathrm{j}}\right)}{1+\mathrm{exp}\left(\upbeta 0+{\upbeta }_{1}{\mathrm{X}}_{1\mathrm{ij}}+{\mathrm{u}}_{0\mathrm{j}}\right)}$$

In another scenario, we might also be interested to see whether the relationship between MCU and an exposure variable ${\mathrm{X}}_{1}$, say wealth tertiles may not be fixed across groups. This would extend a random intercept model to a random coefficient model, by relaxing the assumption that the influence of wealth to MCU is the same across PSUs (villages and streets) and region levels. Thus, the coefficient of variable wealth (${\upbeta }_{1}$) which was initially a fixed effect, should contain random parameter ${\upbeta }_{1}$ is presented as ${\upbeta }_{1}$+${\mathrm{u}}_{01\mathrm{j}}$ and ${\mathrm{u}}_{01\mathrm{j}}$, present a random quantity that allows effect of wealth to be varying across all women’s PSUs of residence. But ${\mathrm{u}}_{0\mathrm{j}}$ and ${\mathrm{u}}_{01\mathrm{j}}$ are assumed to be correlated to estimate the covariance matrix for this correlation and ${\updelta }_{\mathrm{u}01}^{2}$ is the variance associated with random quantity ${\mathrm{ u}}_{01\mathrm{j}}$ for varying coefficient of wealth on the levels of PSUs. Now, consider that the effect of wealth is random across PSUs levels, this extends the simple random intercept model to a random slope model. In general, if we have n covariates ${(\mathrm{X}}_{1},{\mathrm{ X}}_{2}, {\mathrm{X}}_{3, }{\dots ...,\mathrm{X}}_{\mathrm{n}})$, and p other covariates $({\mathrm{X}}_{1},{\mathrm{ X}}_{2}, {\mathrm{X}}_{3, }\dots .{,\mathrm{X}}_{\mathrm{p}}$)*,* associated with varying slope across PSUs on MCU, in typical statistical model with logit-link function this can be expressed as;

9 $$\mathrm{log }\left(\frac{{\uppi }_{\mathrm{ij}}}{{1-\uppi }_{\mathrm{ij}}}\right){=\upbeta }_{0}+{\upbeta }_{\mathrm{z}}{\mathrm{X}}_{\mathrm{zij}}+{\upbeta }_{\mathrm{bj}}{\mathrm{X}}_{\mathrm{bij}}+{\mathrm{u}}_{0\mathrm{j}}+\dots + {\mathrm{u}}_{0\mathrm{bj}}$$

where ${\upbeta }_{\mathrm{bj}}={\upbeta }_{\mathrm{b}}$+${\mathrm{u}}_{0\mathrm{bj}}$ and b = 1,2,3 ……p (number of variables considered as random coefficient, which is wealth for this analysis) and z $=$ 1, 2,3……n (fixed effect parameters associated with covariates which are equal to number of variables under study). Subscripts $\mathrm{n}=9$ which are the selected predictors variables and $\text p=1$ which is for one variable regarded as random coefficient (wealth tertiles).

So far, in the TDHS the PSUs were clustered within regions or provinces creating a further nesting structure. Accordingly, the variance term is partitioned into three more levels, with ${\mathrm{u}}_{0\mathrm{jk}}$ representing a random component associated with the ${\mathrm{i}}^{\mathrm{th}}$ individual in the ${\mathrm{j}}^{\mathrm{th}}$ PSU and the ${\mathrm{k}}^{\mathrm{th}}$ region. So, the respective random parameters are ${\mathrm{e}}_{\mathrm{ij}}$, ${\mathrm{u}}_{0\mathrm{j}}$ and ${\mathrm{u}}_{0\mathrm{k}}$ which gives three levels of variance components for individuals, PSUs and regions with variance terms ${\updelta }_{\mathrm{e}}^{2}, {\updelta }_{\mathrm{u}0}^{2}\mathrm{ and }{\updelta }_{\mathrm{u}1}^{2}$ respectively. The model is given by;

10 $$\mathrm{log }\left(\frac{{\uppi }_{\mathrm{ijk}}}{{1-\uppi }_{\mathrm{ijk}}}\right){=\upbeta }_{0}+{\upbeta }_{\mathrm{z}}{\mathrm{X}}_{\mathrm{zij}}+{\upbeta }_{\mathrm{bj}}{\mathrm{X}}_{\mathrm{bij}}+{\mathrm{u}}_{0\mathrm{j}}+ {\mathrm{u}}_{0\mathrm{bk}}$$

**Fig. 1:**
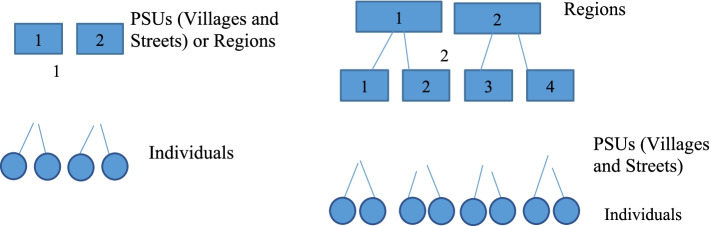
The TDHS data grouping structures two scenarios; (1) Individuals nested within the PSUs or individuals nested within regions (two levels), (2) Individuals are nested within PSUs that are nested in regions (three levels)

Figure 1 shows the nested data structure of TDHS with two levels (left) and three levels (right).

### Analysis

We fitted three weighted generalised linear models to the data, by first starting with a plain model with no covariates but containing random intercepts only. Model 1 includes cluster variable PSU, Model 2 contains cluster variable region, and Model 3 includes both PSU and region. Thus Model 1 and Model 2 are considered as two-level variance component models and Model 3 a three-level variance component model. We included survey weighting in all models. Note that, the analysis of the null model helps in exploring proportions of variance attributed to clustering effects [26, 27]. We also fitted a traditional model for analysing survey data using *svy* command in STATA, Model 0. The best fit model was assessed using Bayesian deviance information criteria (BIC), Akaike information criteria (AIC) and loglikelihood values. Finally, a model with random slopes was constructed to assess variability of wealth tertiles levels on MCU across groups. Models were constructed and fitted using STATA version 16.

## Results

### Background characteristics

Table 1 shows background characteristics of our sample based on TDHS 2015/2016. Out of 5263 individuals included, the sample prevalence of MCU was almost 44% (95% CI: 0.425–0.452). The majority were between the ages of 20–29 and 30–39 years. More than 65% of women had primary education, while 31.36% were from urban areas. Likewise, about 70% of the women had husbands with primary education, 19% secondary and 11% had never been to school.

**Table 1:** Baseline characteristics of unpregnant married women, infecund and with no desire to have children in next 2 years, TDHS 2015/2016

Characteristic	n	% (95% CI)	Modern contraceptive use	
			No, n (%)	Yes, n (%)
Modern contraceptive use				
No	2954	56.13 (54.84; 57.51)		
Yes	2309	43.8 (42.52; 45.22)		
Age group in years				
15–19	289	5.49 (4.89; 6.00)	208 (71.85)	81 (28.15)
20–29	1929	36.65 (35.36; 38.00)	1054 (54.66)	875 (45.34)
30–39	1799	34.19 (32.90; 35.50)	908 (50.47)	891 (49.53)
40–49	1246	23.67 (22.52; 24.81)	746 (59.90)	500 (40.10)
Woman educational levels				
Never	983	18.68 (17.72; 19.83)	649 (65.98)	335 (34.02)
Primary	3512	66.73 (65.40; 68.01)	1865 (53.11)	1647 (46.89)
Secondary +	768	14.59 (13.70; 15.60)	402 (52.42)	366 (47.60)
Parity				
None	21	0.41 (0.25; 0.60)	13 (58.51)	9 (41.49)
One to two	1768	33.59 (32.29; 349)	958 (54.19)	810 (45.81)
Three to four	1717	32.63 (31.37; 33.86)	882 (51.33)	836 (48.67)
Five +	1756	33.37 (32.12; 34.69)	1064 (60.59)	692 (39.41)
Place of resident				
Urban	1650	31.36 (30.13; 32.58)	850 (51.48)	801 (48.52)
Rural	3613	68.64 (67.42; 69.90)	2067 (57.21)	1546 (42.79)
Husband desire for children				
Don’t know	1571	0.41 (028.60; 0.311)	818 (52.05)	753 (47.95)
Both want same	2046	33.59 (37.60; 40.20)	1067 (52.14)	979 (47.86)
Husband desire more	1321	32.63 (23.90; 26.30)	859 (65.01)	462 (34.99)
Husband desire less	325	33.37 (5.60; 6.900)	173 (53.22)	152 (46.78)
Age difference with man				
Woman older than man	212	4.03 (3.50; 4.60)	111 (52.12)	102 (47.88)
Same age level	179	3.41 (3.00; 3.90)	83 (46.29)	96 (53.71)
Man old for 10-	3822	72.66 (71.41;74)	2085 (54.29)	1738 (45.71)
Man old for 10 +	1047	19.9 (18.85; 23.)	636 (60.71)	411 (39.29)
Husband education level				
No education	576	10.94 (10.10; 11.80)	412 (71.64)	164 (28.46)
Primary education	3707	70.44 (69.20; 71.70)	1985 (53.54)	1722 (46.46)
Secondary and higher	980	18.62 (17.61; 19.71)	519 (52.99)	461 (47.01)
Wealth tertiles				
Poor	2001	38.02 (36.72; 39.30)	1323 (66.11)	678 (33.89)
Middle	1031	19.59 (18.52; 20.74)	521 (50.57)	510 (49.43)
Rich	2231	42.42 (41.09; 43.68)	1072 (48.06)	1159 (51.94)
Exposure to media				
Not exposed	893	16.96 (16.03; 18.01)	594 (66.6)	298 (33.4)
Exposed to at least one	1567	29.78 (28.51; 31.04)	951 (60.65)	617 (39.35)
Exposed to at least two	1504	28.57 (27.40; 29.82)	770 (51.19)	734 (48.81)
Exposed to all	1299	24.68 (23.47; 25.85)	601 (46.25)	698 (53.75)

### PSU and regional variations in MCU

Table 2 shows results from three random component models with only random intercepts and dependent variable MCU. Based on all AIC, BIC and log-likelihood values, Model 3 with both PSU and region as clustering variables was supported over Model 1 and Model 2. The AIC and BIC for Model 3 were 6793.758 and 6813.413 smaller than Model 1 and Model 2. Also, Model 3 had a bigger log-likelihood value compared to all models which implies there was both PSU and regional variabilities on MCU. Similarly, the Model 1 had a better fit than Model 2, meaning that there was substantially more variance in MCU at PSU level than at regional level. Model 0 for the traditional survey modelling approach had the lowest loglikelihood value of 3664.23 compared to all.

**Table 2:** Traditional, two level and three level empty random components models for MCU

Parameter	Model 0 (Traditional)	Model 1 (PSU)	Model 2 (Region)	Model 3 (PSU and Region)
${\updelta }_{\mathrm{u}0}^{2}$(Variance ${\mathrm{u}}_{0\mathrm{j}}$)	-	0.74 (0.308; 0.566)		0.47 (0.308; 0.566)
${\updelta }_{\mathrm{u}1}^{2}$(Variance ${\mathrm{u}}_{0\mathrm{k}}$)	-		0.35 (0.22; 564)	0.39 (0.267; 0.849)
ICC(Region)	-		0.10 (0.063; 0.146)	0.09 (0.058;147)
ICC(PSU)	-	0.18 (0.149; 0.224)		0.21 (0.160; 0.261)
AIC	7330.46	6885.68	6961.31	6793.76
BIC	7337.011	6898.78	6974.41	6813.41
Loglikelihood	-3664.23	-3440.84	-3478.66	-3393.88

In Model 3, it was found that the PSU level variance (${\updelta }_{\mathrm{u}0}^{2}=$) 0.466 was bigger than the regional level variance ${(\updelta }_{\mathrm{u}1}^{2}=$) 0.387. This implies that there was significant PSU level variability than region level variability on MCU. The intra-class correlation coefficients (ICCs) which is a measure for the relatedness of individuals within a same group was 0.205 for PSU and 0.094 for region. That means, about 21% and 9% variations in MCU among WRA in Tanzania were attributable to clustering at PSU and region, respectively.

### Determinants of modern contraceptive use accounting for variance structures

Following inclusion of wealth tertiles in the model, MCU was 95% more likely in the age group 20–29 years than in 15–19 years (OR = 195; 95% CI: 1.261–3.009). WRA aged 30–39 years were more than twice as likely to use modern contraceptives methods than those aged 15–19 years (OR = 2.29; 95% CI: 1.379 -3.809). WRA exposed to at least radio or television or newspapers, and women with exposure to other two or three media were 5%, 38% and 54% more likely to use modern contraceptives than women with no exposure to media. Similarly, after adjusting for PSU and region clusters and random variation of wealth tertiles, the odds of MCU among middle and rich WRA were 1.60 (95% CI: 1.284–1.987) and 2.18 (95% CI: 1.632–2.923) times the odds among WRA in the poor wealth tertiles. WRA with husbands who desired to have more children were 65% less likely to use modern contraceptive (Table 3). Women who had primary, and secondary + education were 20% and 6% more likely to use modern contraceptives than women who had never been to school, but this was statistically insignificant.

**Table 3:** Variance components model with a random intercepts and random coefficient examining the determinants of MCU, TDHS, 2015/2016

Fixed part	Random intercept Model 3	Random coefficient Model 3
	OR (95%CI)	OR (95%CI)
Age in years groups		
15–19		
20–29	1.94 (1.244;3.024)	1.95 (1.261; 3.009)
30–39	2.28 (1.372; 3.803)	2.29 (1.379; 3.809)
40–49	1.41 (0.819; 2.432)	1.40 (0.810; 2.409)
Woman educational levels		
Never		
Primary	1.22 (0.985; 1.507)	1.20 (0.963; 1.488)
Secondary +	1.08 (0.776; 1.497)	1.06 (0.765; 1.477)
Parity		
None		
One to two	1.39 (0.471; 4.111)	1.39 (0.464; 4.140)
Three to four	1.45 (0.484; 4.335)	1.45 (0.478; 4.383)
Five +	1.40 (0.443; 4.403)	1.39 (0.436; 4.454)
Place of resident		
Urban		
Rural	1.10 (0.848; 1.429)	1.12 (0.863; 1.442)
Husband desire for children		
Don’t knows		
Both want same	0.82 (0.691; 0.972)	0.82 (0.686; 0.972)
Husband desire more	0.64 (0.489; 0.850)	0.65 (0.493; 0.865)
Husband desire less	0.84 (0.644; 1.097)	0.83 (0.630; 1.098)
Age difference with man		
Woman older than man		
Same age level	1.16 (0.779; 1.717)	1.17 (0.780; 1.763)
Man old for 10-	0.83 (0.646; 1.065)	0.83 (0.642; 1.070)
Man old for 10 +	0.73 (0.536; 1.003)	0.73 (0.535; 1.006)
Husband education level		
No education		
Primary education	1.58 (1.232; 2.019)	1.52 (1.174; 1.980)
Secondary and higher	1.36 (0.929; 1.981)	1.34 (0.921;1.939)
Wealth tertiles		
Poor		
Middle	1.57 (1.260; 1.949)	1.60 (1.284;1.987)
Rich	2.14 (1.617; 2.826)	2.18 (1.632; 2.923)
Exposure to media		
Not exposed		
Exposed to at least one	1.05 (0.763; 1.451)	1.05 (0.759; 1.445)
Exposed to at least two	1.39 (1.091; 1.770)	1.38 (1.087; 1.747)
Exposed to all	1.54 (1.189; 2.002)	1.54 (1.187; 1.988)
Random part		
${\updelta }_{\mathrm{u}0}^{2}=$ Variance ${ (\mathrm{u}}_{0\mathrm{j})}$)	0.360 (0.193; 0.671)	0.718 (0.413;1.248)
$${\updelta }_{\mathrm{u}1}^{2}$$$$=$$ Variance ($${\mathrm{u}}_{0\mathrm{k}}$$)	0.341 (0.215; 0.541)	0.381 (0.191; 0.762)
${\delta }_{u01}^{2}=\mathrm{Var}-\mathrm{Cov}$(${\mathrm{u}}_{01, }{\mathrm{u}}_{0\mathrm{j}}$)		0.137 (0.049; 0.377)
${\updelta }_{\mathrm{u}11}^{2}=\mathrm{Var}-\mathrm{Cov}$(${\mathrm{u}}_{11, }{\mathrm{u}}_{0\mathrm{k}}$)		0.022 (0.002; 0.206)
ICC (region)	0.090 (0.050; 0.156)	0.087 (0.045; 0.161)
ICC (PSU)	0.176 (0.127; 0.237)	0.251 (0.179; 0.338)
AIC	6590.534	6577.618
BIC	6754.309	6761.046
Loglikelihood	-3270.2668	-3260.809

### The effect of additional random covariate on MCU

A random coefficient Model 3 was fitted to data to assess the influence of wealth on MCU as a random, not fixed effect (Table 3). The loglikelihood values for Model 3 with the random coefficient and Model 3 with random intercepts were -3260.809 and -3270.267 respectively, indicating significant importance to retain a random coefficient variable to the model. That is, Model 3 with random coefficients fitted the data better than Model 3 without a random coefficient variable. The variance covariance between wealth tertiles and region was 0.022, implying there was positive heterogeneity for wealth on MCU across regional levels by 2%. Likewise, the variance covariance for wealth tertiles and PSU was 0.137, meaning that there was nearly 14% significant variation of wealth tertiles level on MCU across PSUs level (${\updelta }_{\mathrm{u}01}^{2}=$ 0.137; 95% CI: 0.49–0.377). However, while adding a random coefficient component for wealth tertiles improved goodness of fit, a woman’s educational level remained insignificantly associated with MCU. Also, there were significant increase in the ICC values for PSU from 17.6% to 25.4% and reduction of those for region from 9 to 8% when comparing the two respective models (random intercept vs random slope model). In general, age of a woman, education level of a man, desire to have more children, exposure to media and wealth tertiles are the significant covariates for MCU among WRA in Tanzania.

### Strengths and limitations

Given the nature of cross-sectional study design; similarly, for this study, it was challenging in making casual inferences due to difficulty in determining the sequence of occurrence between set of selected exposure variables such as age, woman educational level, and outcome of interest (MCU). This study was not able to capture characteristics relating to knowledge, attitude, and practices of MCU in Tanzania.

The main strength of TDHS data is representation of the entire population of the WRA in the country and the nested data structure. With the nested data structure of TDHS, this study was able to consider respective associated variance structures in relation to determinants of MCU uptake.

## Discussion

We used a variance components modelling approach to exploit the structure of the TDHS data in addressing MCU. We explored various techniques including analysis of simple models then extending to a random covariate model. We identified significant fixed and random effects influencing MCU. With fixed effects, age of a woman, a woman’s husband education levels, exposure to media, wealth tertiles and desire to have more children are the important covariates significantly associated with MCU among WRA in Tanzania. We also found presence of random effects in forms of both random intercepts and slopes.

Analysis of random intercept model, an empty random component Model 3 showed evidence for variations in MCU at PSU and region levels. The significance of random intercepts compared to traditional models was also reported by many authors [10, 11, 28–30]. Greater variation was attributable to PSUs (25%) than regions (about 10%) and clustering extended to the analysis of a covariate model (Model 3 with random slopes). This could reflect the existence of an effect of neighbourhood or grouping effect more strongly associated with PSU clustering than region, perhaps because accessing modern contraceptives from health facilities is easier in more urban than rural areas. Characteristics like wealth and education levels may also vary between villages and streets corresponding more closely with the scale of PSUs than regions. Studies from Nigeria, Zimbabwe and Ethiopia have reported positive influence of community levels on MCU estimated at PSU level [10, 11, 31]. However, most of these studies were conducted with only one cluster variable (PSU), which limits the comparability of their results with ours. The 10% regional variation could also represent structural provision of MCU services at regional levels. Studies have suggested the presence of effects of neighbourhood or community on health related outcomes that is measured at PSU level (the lower level cluster) in our study setting [32–34]. On the other hand, the ICCs for PSU was larger than ICC for region, perhaps because there was smaller number of individuals at region than PSU clusters as the ICCs were reported to be inversely related with cluster size as presented in [9].

As far as group effects on MCU, the effects of covariates are most notable when fixed effects were introduced to an empty variance component model and when more random parameters are added to the models. Ferede and Ejembi [10, 11] show changes in values of random intercepts as a result of adding covariates during model selection [10, 11]. However, covariates such as age of a woman, desire to have more children, wealth tertiles, and media exposure remained important determinants of MCU [35–38]. In the desire to have more children in the future, when men desire more children WRA are less likely to use modern contraceptives and this could possibly imply men have greater power to decide on family planning services in many Tanzanian families. In this study, WRA in middle and rich wealth tertiles and those who are more exposed to media outlets have greater odds of using MCU than their counterparts. This could mean that wealthier households with WRA have more access to media sources. Also, because most of these characteristics were significant in past studies [6, 10, 11, 19, 20], some similarity in findings was expected in this study. We also found that woman educational level is insignificantly associated with MCU, which is similar to [38].

## Recommendations for public health policy and research practises

There is a considerable variation in MCU due to hierarchical structure of the population at PSU and regional levels. We illustrated that it is possible for population characteristics such as levels of wealth tertiles to have random influences on MCU from one population hierarchy to another. This highlights the presence of dynamics in populations characteristics and their effect on health outcomes such us MCU. For public health policy, the implications are that health policy makers should be aware of between group variations (PSUs and regions) with respect to MCU uptake. This is because such variational impacts not only on MCU but also on other characteristics of public health importance in the country. The random effects of wealth tertiles across clusters reflect disparities on wealth levels between regions and PSUs on MCU, that requires more attention in the allocation of family planning services in Tanzania. Promoting family planning services using media such as radio and televisions and women empowerments on MCU in the community requires great considerations.

Furthermore, after taking into consideration the nested data structure and associated random quantities, we found different estimates between models and the overall best fit model. For example, when two levels of variance structure fitted, results differed from when three level were fitted to data. Also, the parameter estimates from the model with random intercepts differed from the model with both random intercept and random slopes pointing to the need for statisticians to be careful in the selection of appropriate modelling techniques with respect to the data structure. However, because this is survey-based data, it is common to use survey modelling approaches but since they give no representation to any form of random effects parameters various variance structures associated in the data would not be captured. This study shows how robust modelling methods for clustering that reflect the hierarchical relationships of variance structure are important for accurate inference.

## Conclusion

Here we highlight the use of a variance components modelling approach as a promising gold standard in analysing DHS data by considering multiple nested levels. We suggest future studies should consider more hierarchy levels associated with DHS data, although it may become more complicated extending to additional variance components. While high prevalence of modern contraceptive use is recognised in fertility control, prevalence is still low in Tanzania leading to higher fertility rate. This study highlights the existence of village and street and region variabilities, and their group level influence on MCU. Also, the study highlights the significant differences of wealth textiles across PSUs and regional levels that should be considered to ensure more equitable allocation of family planning intervention between population levels.

## Data Availability

DHS datasets are publicity available at the DHS website; www.dhsprogram.com.

## Abbreviations

DHS: Demographic and health survey
ICC: Intra-cluster correlations coefficient
MCU: Modern contraceptive use
PSU: Primary sampling unit
TDHS: Tanzania Demographic and Health Survey
WRA: Women of reproductive age

## References

[1] World Health Organisation. Reproductive health indicators: guidelines for their generation, interpretation and analysis for global monitoring. WHO Publ Geneva. 2006. 10.1016/S0277-9536(02)00341-6.

[2] Darmoul D, Baricault L, Sapin C, Chantret I, Trugnan G, Rousset M. Health in 2015 from MDGs millennium development goals to SDGs sustainable development goals. Geneva: World Heal Organization; 2015. https://apps.who.int/iris/handle/10665/200009.

[3] United Nations, Department of Economic and Social Affairs PD (2015). Trends in contraceptive use worldwide 2015. Contraception.

[4] World Health Organization. WHO progress report. Reproductive health strategy to accelerate progress towards the attainment of international development goals and targets. Geneva: World Health Organization; 2004. 10.1016/s0968-8080(05)25166-2 16035592

[5] Singh S, Darroch EJ. Adding it up: costs and benefits of contraceptive services estimates for 2012. Guttmacher Inst United Nations Popul Fund (UNFPA); 2012. p. 201. Available online at http://www.guttmacher.org/pubs/AIU-2012-estimates.pdf.

[6] The United Republic of Tanzania Ministry of Health and Social Welfare. The national family planning costed implementation program. Dar es salaam: The Ministry of Health and Social Welfare (MoHSW); 2013.

[7] Smith R, Ashford L, Gribble J, Clifton D. Family planning saves lives. 4th ed. Washington DC: Population Reference Bureau; 2009.

[8] Price. Addressing the reproductive health needs and right of young people since ICPD- the contribution of UNFPA and IPPF. Synthesis report. Euro Health Group. University of Heidelberg; 2004.

[9] Ministry of Health, Community Development, Gender, Elderly and Children (MoHCDGEC) [Tanzania Mainland], Ministry of Health (MoH) [Zanzibar], National Bureau of Statistics (NBS), Office of the Chief Government Statistician (OCGS) and ICF. Tanzania demographic and health survey and malaria indicator survey (TDHS-MIS) 2015–16. Dar es Salaam and Rockville: MoHCDGEC, MoH, NBS, OCGS, and ICF; 2016.

[10] Ferede T (2013). Multilevel modelling of modern contraceptive use among rural and urban population of Ethiopia. Am J Math Stat.

[11] Ejembi CL, Dahiru T, Aliyu AA (2015). Contraceptive use in Nigeria. DHS Work. Pap. No. 120.

[12] Kidayi PL, Msuya S, Todd J, Mtuya CC, Mtuy T, Mahande MJ (2015). Determinants of modern contraceptive use among women of reproductive age in Tanzania: evidence from Tanzania demographic and health survey data. Adv Sex Med.

[13] Exavery A, Mubyazi GM, Rugemalila J (2012). Acceptability of condom promotion and distribution among 10–19 year-old adolescents in Mpwapwa and Mbeya rural districts, Tanzania. BMC Public Health.

[14] Somba MJ, Mbonile M, Obure J, Mahande MJ (2014). Sexual behaviour, contraceptive knowledge and use among female undergraduates’ students of Muhimbili and Dar es Salaam Universities, Tanzania: a cross-sectional study. BMC Womens Health.

[15] Kashagam E, Ngocho JS (2015). Prevalence of modern contraceptive methods use among women living with HIV attending care and treatment clinic at Amana Hospital Dar Es Salaam, Tanzania. Int J Soc Sci Humanit Invent.

[16] Mwangeni AE, Ankomah A, Richard AP (1998). Attitudes of men towards family planning in Mbeya region, Tanzania: a rural – urban comparison of qualitative data. J Biosoc Sci.

[17] Carle AC (2009). Methodology fitting multilevel models in complex survey data with design weights: recommendations. BMC Med Res Methodol.

[18] ICF. Demographic and health surveys standard recode manual for DHS7. The Demographic and Health Surveys Program. Rockville: ICF; 2018.

[19] Stephenson R, Baschieri A, Clements S, Hennink M, Madise N (2007). Contextual influences on modern contraceptive use in sub-Saharan Africa. Am J Public Health.

[20] Lasong J, Zhang Y, Gebremedhin SA, Opoku S, Abaidoo CS, Mkandawire T, Zhao K, Zhang H (2020). Determinants of modern contraceptive use among married women of reproductive age: a cross-sectional study in rural Zambia. BMJ Open.

[21] Sahai H, Ojeda MM (2005). Analysis of variance for random models. Anal Var Random Model.

[22] Merlo J. Multilevel modelling of health statistics: Edited by A H Leyland, H Goldstein. New York: Wiley, 2001. J Epidemiol Community Heal. 2002. 10.1136/jech.56.7.560-a.

[23] Diez-roux AV (2000). Multilevel analysis in public health research. Annu Rev Public Health.

[24] Roux AVD (2002). A glossary for multilevel analysis. J Epidemiol Community Health.

[25] Zuur A, Ieno EN, Walker N, Saveliev A, Smith GM. Mixed effects models and extensions in ecology with R. 2009. 10.1007/978-0-387-87458-6_1.

[26] De LJ, Meijer E (2008). Introduction to multilevel analysis. Handb Multilevel Anal.

[27] Hox JJ, Moerbeek M, van de Schoot R. Multilevel analysis: techniques and applications, second edition. New York: Routledge; 2010. 10.4324/9780203852279.

[28] Makupe DJ, Kumwenda S, Kazembe L (2019). An application of mixed-effect models to analyse contraceptive use in Malawian women. Contracept Reprod Med.

[29] Wulifan JK, Brenner S, Jahn A, De Allegri M (2015). A scoping review on determinants of unmet need for family planning among women of reproductive age in low and middle income countries. BMC Womens Health.

[30] Dias JG, De Oliveira IT. Multilevel effects of wealth on women’s contraceptive use in Mozambique. PLoS ONE. 2015;10(3):1–15. e0121758. 10.1371/journal.pone.0121758. 10.1371/journal.pone.0121758 PMC4364712 25786228

[31] Mcguire C, Stephenson R (2015). Community factors influencing birth spacing among married women in Uganda and Zimbabwe. Afr J Reprod Health.

[32] Gary-Webb TL, Baptiste-Roberts K, Pham L, Wesche-Thobaben J, Patricio J. Neighborhood and weight-related health behaviors in the Look AHEAD (Action for Health in Diabetes) study. 2010. 10.1186/1471-2458-10-312. 10.1186/1471-2458-10-312 PMC2897795 20525373

[33] Kruk ME, Rockers PC, Mbaruku G, Paczkowski MM, Galea S (2010). Community and health system factors associated with facility delivery in rural Tanzania: a multilevel analysis. Health Policy (New York).

[34] Belachew AB, Kahsay AB, Abebe YG (2016). Individual and community-level factors associated with introduction of prelacteal feeding in Ethiopia. Arch Public Health.

[35] Janevic T, Sarah PW, Leyla I, Elizabeth BH (2012). Individual and community level socioeconomic inequalities in contraceptive use in 10 Newly Independent States: a multilevel cross-sectional analysis. Int J Equity Health.

[36] Vu LTH, Oh J, Bui QT-T, Le AT-K (2016). Use of modern contraceptives among married women in Vietnam: a multilevel analysis using the Multiple Indicator Cluster Survey (2011) and the Vietnam Population and Housing Census (2009). Glob Health Action.

[37] Kaggwa EB, Diop N, Douglas J (2008). The role of individual and community normative factors: a multilevel analysis of contraceptive use among women in union in Mali. Int Fam Plan Perspect.

[38] Ngome E, Odimegwu C (2014). The social context of adolescent women’s use of modern contraceptives in Zimbabwe: a multilevel analysis. Reprod Health.

