# Correction: Application of variance components to the identification of determinants of modern contraceptive use in the Tanzania demographic and health survey data

**DOI:** 10.1186/s12889-022-13813-6

**Published:** 2022-08-01

**Authors:** Oliva Safari Donni, Dunstan Raphael Bishanga, Isambi Sailon Mbalawata

**Affiliations:** 1 Department of Monitoring, Medical Teams International (MTI) Tanzania, Learning and Evaluation, P O Box 1, Kibondo, Tanzania; 2 School of Public Health and Social Sciences, Muhimbili University of Health and Allied Sciences, P O Box, 65001 Dar es Salaam, Tanzania; 3 AIMS-NEI Global Secretariat, Gasabo Kacyiru – Kamatamu, KG590, ST Kigali, Rwanda

**Correction: BMC Public Health 22, 1291 (2022)**

**https://doi.org/10.1186/s12889-022-13,636-5**

Figure 1 in the original publication of this article [1] was distorted. The updated figure is shown in this correction article, the original article has been updated.

**Fig. 1:**
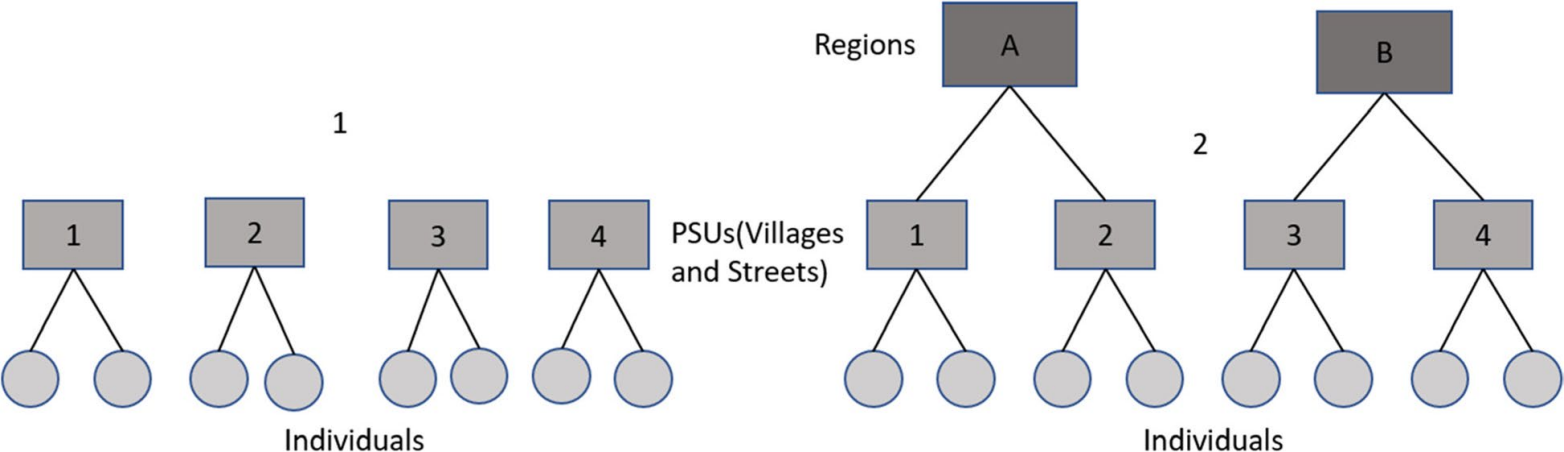
The TDHS data grouping structures two scenarios; (1) Individuals nested within the PSUs or individuals nested within regions (two levels), (2) Individuals are nested within PSUs that are nested in regions (three levels)
